# In vitro activity of Schinus terebinthifolius extract and fractions against Sporothrix brasiliensis

**DOI:** 10.1590/0074-02760220063

**Published:** 2022-09-30

**Authors:** Fernando Almeida-Silva, Andrea Reis Bernardes-Engemann, Ana Luiza Rangel Bérenger, Vagner Pereira da Silva, Maria Raquel Figueiredo, Dayvison Francis Saraiva Freitas

**Affiliations:** 1 Fundação Oswaldo Cruz-Fiocruz, Instituto Nacional de Infectologia Evandro Chagas, Laboratório de Micologia, Rio de Janeiro, RJ, Brasil; 2 Fundação Oswaldo Cruz-Fiocruz, Instituto de Tecnologia em Fármacos, Laboratório de Química de Produtos Naturais, TecBio/LDFito, Far-Manguinhos, Rio de Janeiro, RJ, Brasil; 3 Fundação Oswaldo Cruz-Fiocruz, Instituto Nacional de Infectologia Evandro Chagas, Laboratório de Pesquisa Clínica em Dermatologia Infecciosa, Rio de Janeiro, RJ, Brasil

**Keywords:** Sporothrix brasiliensis, sporotrichosis, antifungal agents, fungal drug sensitivity tests, Schinus terebinthifolius

## Abstract

**BACKGROUND:**

*Sporothrix brasiliensis* is the causative agent of zoonotic cases of sporotrichosis in Brazil and is associated with atypical and severe presentations in cats, dogs, and humans. Sporotrichosis treatment is usually time- and cost-consuming, sometimes with poor response and host toxicity. *Schinus terebinthifolius* has proven efficacy against bacteria and fungi of clinical interest.

**OBJECTIVE:**

To determine the *in vitro* activity of *S. terebinthifolius* against *S. brasiliensis*.

**METHODS:**

Five *S. brasiliensis* isolates and three reference strains were subjected to a hydroethanol extract derived from the leaves of *S. terebinthifolius* and its fractions. The minimal inhibitory concentration (MIC) was determined using the broth microdilution method according to the M38-A2 CLSI guidelines. Also, the fungicidal/fungistatic activity of the extract and fractions was studied.

**FINDINGS:**

The crude extract of *S. terebinthifolius* inhibited the growth of *S. brasiliensis* (MIC: 0.5-1.0 µg/mL), while the partitioned extracts dichloromethane, ethyl acetate, and butanol demonstrated growth inhibition at 8 µg/mL due to a fungistatic activity.

**MAIN CONCLUSIONS:**

Due to its *in vitro* efficacy against *S. brasiliensis* and its known pharmacological safety, *S. terebinthifolius* is a candidate to be tested using *in vivo* models of sporotrichosis.

The genus *Sporothrix* comprises at least 50 species. Although, most of them are described as saprobiotic organisms and found in decaying organic matter,^1^ ^,^ ^2^ three species, *Sporothrix brasiliensis* [Supplementary data (Fig. 1)], *Sporothrix schenckii*, and *Sporothrix globosa*, are of main clinical importance worldwide.^3^

These thermo-dimorphic human pathogens are usually introduced by traumatic inoculation and can cause sporotrichosis,^4^ the main subcutaneous mycosis in Brazil.^5^ At present, the most reported traumatic inoculation route in Brazil is related to scratches and bites from infected cats [Supplementary data (Fig. 2A)].^2^ However, there are still reports of non-zoonotic inoculation in several other countries. The spectrum of the disease can be related to the immune status of the individual, fungal load, virulence, and the depth of traumatic inoculation [Supplementary data (Fig. 2B-C)]. Sporotrichosis is usually benign, localised, and restricted to the skin and the adjacent lymphatic vessels. Nevertheless, people with immunosuppressive conditions, such as those living with human immunodeficiency virus/acquired immunodeficiency syndrome (HIV/AIDS), may present with disseminated disease, including bone, pulmonary, meningeal, and bone marrow involvement. In immunocompetent patients, the disease can also present a severe clinical picture. Recently, sporotrichosis has also been associated with a socially excluded population, notably in the context of zoonotic transmission in poorly structured urban areas.^6^

Sporotrichosis treatment depends mainly on the clinical form of the disease and immunological status of the host. According to some antifungal studies, the species involved in this infection seems to be a reasonable concern.^7^ The main drugs used for treatment of sporotrichosis are itraconazole, potassium iodide, terbinafine, and amphotericin B.^8^ Drug repositioning studies using the Medicines for Malaria Venture (MMV) Pathogen Box^®^ have demonstrated potential antifungal compounds against several fungal agents,^9^ ^,^ ^10^ including *S. brasiliensis* and *S. schenckii*.^11^

In recent decades, many medicines derived from natural products have been introduced for the treatment of various diseases. Plants remain effective in innovative biological approaches. These approaches can be directed towards the development of medicines based on natural products of plant origin.^12^ *S. terebinthifolius* Raddi, popularly known as pepper tree, belongs to the Anacardiaceae family (Fig. 1). This species is largely found on the Brazilian coast and is distributed from the northeast to the southern parts of the country.^13^ *S. terebinthifolius* is included on RENISUS (Brazilian National List of Medicinal Plants of Interest to the United Health System).^14^ Folk medicine is used to treat ulcers, respiratory problems, wounds, rheumatism, gout tumours, diarrhoea, inflammatory events, skin diseases, and arthritis. Several related activities have been confirmed in scientific studies.^15^ ^,^ ^16^ ^,^ ^17^ The antimicrobial activity of *S. terebinthifolius* extracts has been demonstrated, mostly in species of the genus *Candida*, *Leishmania* spp. and *Pseudomonas aeruginosa.* ^18^ The fingerprint of *S. terebinthifolius* shows the presence of chemical classes of phenols, tannins, steroidal saponins, sterols, terpenes, flavonoids and biflavonoids.^19^ ^,^ ^20^ ^,^ ^21^ ^,^ ^22^ The literature shows that compounds isolated from *S. terebinthifolius*, such as luteolin, quercetin, kaempferol, agathisflavone, gallic acid, methyl gallate, 1,2,3,4,6-pentagalloylglucose, epicatechin, coumaric acid, and myricetrin, are part of the chemical profile of the species (Fig. 2).^17^ ^,^ ^18^ ^,^ ^19^ ^,^ ^20^ ^,^ ^22^

The present investigation aimed to determine the *in vitro* activity of *S. terebinthifolius* against *S. brasiliensis*, the main agent of zoonotic sporotrichosis in Brazil.

**Fig. 1:**
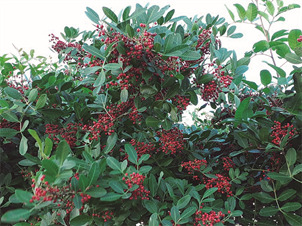
tree of Schinus terebinthifolius Raddi.

**Fig. 2:**
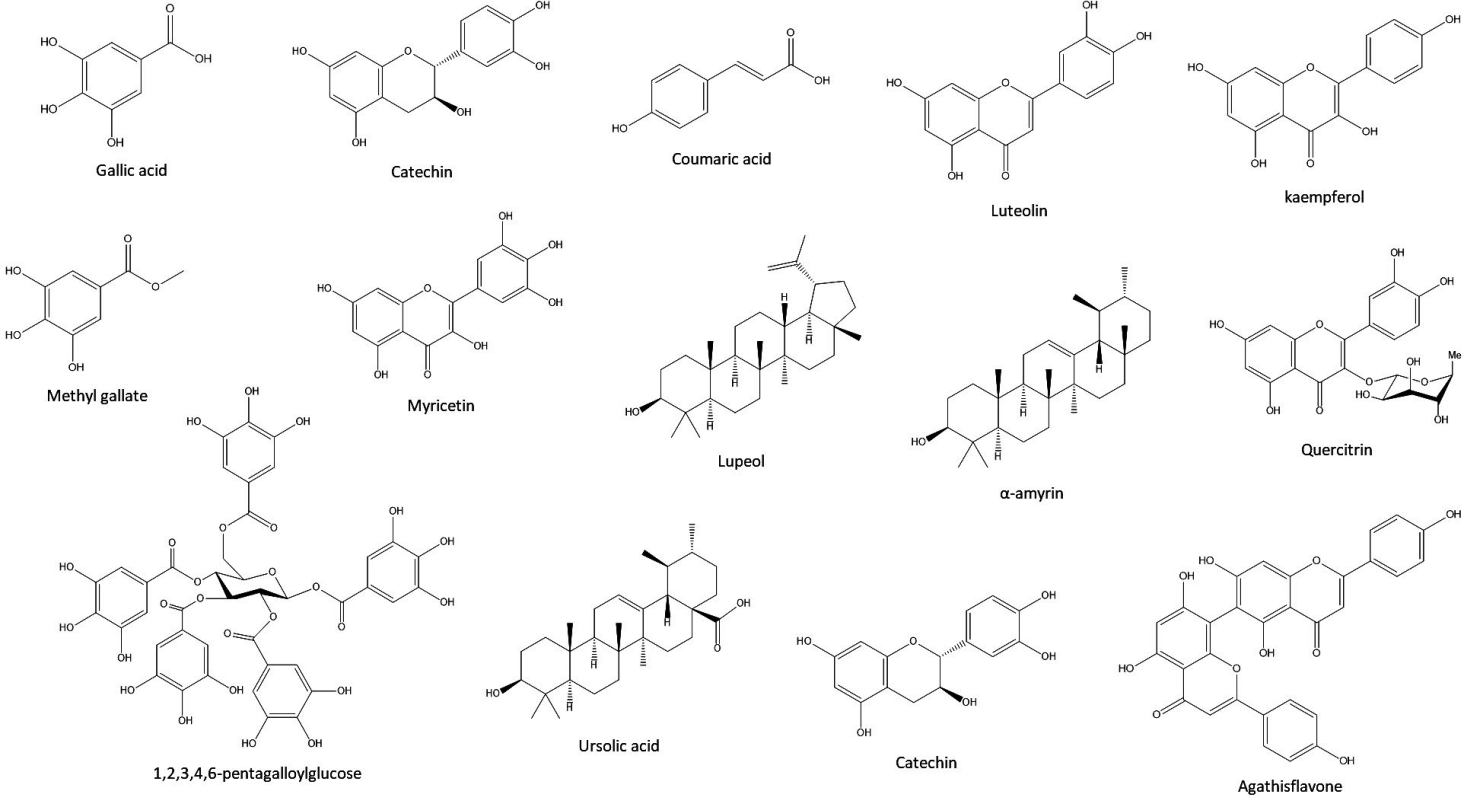
the chemical profile of *Schinus terebinthifolius* Raddi.

## MATERIALS AND METHODS

*Plant material, extract, and fractions* - The leaves of *S. terebinthifolius* Raddi were collected from the campus of the Oswaldo Cruz Foundation (FIOCRUZ), Rio de Janeiro, Brazil. A voucher of this plant was deposited at the Rio de Janeiro Botanical Garden Herbarium (RB) under the number RB 451742 (http://rb.jbrj.gov.br/v2/regua/visualizador.php?r=true&colbot=rb&codtestemunho=00494089&arquivo=00494089.dzi). The species is registered in the Genetic Patrimony (CGEN) under the number AB5D582.

The plant material was dried at room temperature (25-30ºC), reduced to small fragments, and subjected to dynamic extraction using 70% ethanol for 72 h. The extract was filtered, concentrated under reduced pressure, and lyophilised to obtain the crude hydroethanol extract (STFE70). All steps were performed at room temperature.

**Fig. 3:**
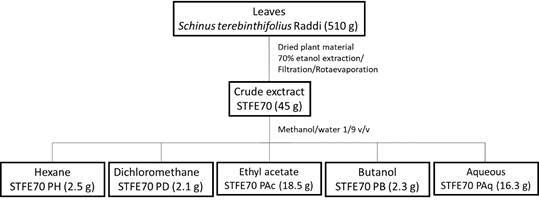
the liquid-liquid partition of the *Schinus terebinthifolius* crude extract.

To perform the liquid-liquid partition procedure, the crude extract (STFE70) was solubilised in methanol/water (1/9 v/v). This hydroalcoholic solution was sequentially fractionated using solvents with increasing polarities: hexane (STFE70 PH), dichloromethane (STFE70 PD), ethyl acetate (STFE70 PAc), and butanol (STFE70 PB). In addition to the four fractions mentioned above, a fifth fraction of wastewater/aqueous (STFE70 PAq) was obtained (Fig. 3). The fractions resulting from the partition techniques and the aqueous residue were concentrated using a rotary evaporator and lyophilised.

*Microorganisms and culture conditions* - Five isolates, IPEC 45434-1 (isolate 1), IPEC 49240 (isolate 2), IPEC 49263 (isolate 3), INI 50639-1 (isolate 4) and INI 50659 (isolate 5), obtained from the Collection of Pathogenic Fungi of the Evandro Chagas National Institute of Infectious Diseases, FIOCRUZ, and identified as *S. brasiliensis* by a protocol previously described,^23^ were included, in addition to three reference strains used in the experiments, *S. brasiliensis* - CBS 120339 (isolate 6), *S. schenckii* - IPEC 36277 (isolate 7) and *S. globosa* - IPEC 27135 (isolate 8). All isolates were cultured on potato dextrose agar (PDA) (Sigma Chemical Corporation, St. Louis, MO, USA) for seven days at 35ºC for minimal inhibitory concentration (MIC) assays.

*MIC assays and determination of minimal fungicidal concentration (MFC)* - After incubation of each isolate in PDA for seven days at 35ºC, inocula of 1-5 x 10^4^ conidia/mL were prepared in sterile saline solution and diluted in RPMI-1640 medium (Sigma-Aldrich) (pH 7.0) with 0.165 mol/L morpholinepropanesulfonic acid (MOPS) to perform the broth microdilution method according to the M38-A2 CLSI guidelines.^24^ Different concentrations (0.015-8 µg/mL) of *S. terebinthifolius* plant extracts were distributed into 96-well microplates, and the MIC was determined by the lowest concentration able to inhibit fungal growth. Growth controls were determined in wells containing RPMI-1640 medium with 1% dimethyl sulfoxide (DMSO) and fungal inoculum without any *S. terebinthifolius* extracts, and wells containing only medium and DMSO were used as sterility controls. MIC determination was performed by visual inspection after 72 h of incubation at 35ºC as previously described.^25^ The MFC was determined by transferring an aliquot of 5 μL of each well without fungal growth from the microplates used for MIC to 96-well microplates with Sabouraud 2% glucose agar (Sigma Chemical Corporation). The compound activity was determined as the lowest drug concentration without fungal growth on Sabouraud 2% glucose agar after five days of incubation at 35ºC. When the MFC/MIC ratio of a compound was 1 or 2, the substance was considered fungicidal against the pathogen and, if the ratio was greater than 2, the probable mode of action was fungistatic.^26^

## RESULTS AND DISCUSSION

The phytochemical strategy with liquid-liquid partition of *S. terebinthifolius* extract using different organic solvents with increasing polarity gradients allowed the separation of chemical classes and improved the concentration of compounds that could be responsible for the biological activity. The 45 g of the crude extract (STFE70) led to fraction yields of 5.6% (STFE70 PH), 4.7% (STFE70 PD), 41.1% (STFE70 PAc), 5.1% (STFE70 PB) and 36.2% (STFE70 PAq) (Fig. 3).

The MIC of STFE70 (crude extract) for all *S. brasiliensis* isolates and *S. schenckii* ranged from 0.5 to 1 µg/mL, and the MIC for *S. globosa* was obtained at 0.25 µg/mL. The antifungal susceptibility test with the partitioned extracts, STFE70 PD, STFE70 PAc, and STFE70 PB demonstrated growth inhibition at 8 µg/mL for all isolates. STFE70 PH and STFE70 PAq demonstrated no activity up to 8 µg/mL. All extracts that inhibited fungal growth demonstrated fungistatic activity with values higher than 2 and individual results are summarised in Table.

**TABLE:** Determination of the minimal inhibitory concentration (MIC) of *Schinus terebinthifolius* extract and fractions against *Sporothrix brasiliensis*

*Sporothrix* sp. isolates		*S. terebinthifolius* extract and fractions - MIC (µg/mL)	MFC/MIC
		STFE70		STFE70 PH	STFE70 PD	STFE70 PAc	STFE70 PB	STFE70 PAq
	1	1	No activity	8	8	8	No activity	8
	2	0.5	No activity	8	8	8	No activity	16
	3	1	No activity	8	8	8	No activity	8
	4	0.5	No activity	8	8	8	No activity	16
	5	0.5	No activity	8	8	8	No activity	16
	6	0.5	No activity	8	8	8	No activity	16
	7	1	No activity	8	8	8	No activity	8
	8	0.25	No activity	8	8	8	No activity	32

Isolates: *S. brasiliensis* (1 - IPEC 45434-1; 2 - IPEC 49240; 3 - IPEC 49263; 4 - INI 50639-1; 5 - INI 50659). 6 - CBS 120339 (*S. brasiliensis* reference strain). 7 - IPEC 36277 (*Sporothrix schenckii* reference strain). 8 - IPEC 27135 (*Sporothrix globosa* reference strain). MIC: minimal inhibitory concentration; STFE70: crude hydroethanol extract; Fractions: STFE70 PH - hexane; STFE70 PD - dichloromethane; STFE70 PAc - ethyl acetate; STFE70 PB - butanol; STFE70 PAq - wastewater/aqueous. Minimal fungicidal concentration (MFC)/MIC - represents the ratio calculated with the values obtained in the minimal fungicidal concentration experiment and the STFE70 MIC values.

The number of microorganisms resistant to conventional treatments has been increasing. Several aspects can influence this resistance, such as intrinsic mechanisms, use of pesticides, and the indiscriminate use of antibiotic/antifungal drugs. The main resistance mechanisms described for the genus *Sporothrix* include melanin production capacity, genetic diversity, and mutations in cytochrome P450.^27^ Some isolates that are intrinsically resistant to traditional antifungal agents have been reported previously, and based on their susceptibility profiles, it is possible to classify the isolates as wild type and non-wild type; however, this classification is used only for conventional antifungal drugs.^7^ On the other hand, there are few unconventional treatment options for sporotrichosis, which can include cryosurgery and thermotherapy in pregnant women and in cases of contraindication or intolerance to conventional antifungal drugs.^28^

Thus, the search for new molecules and repositioning studies is increasing and demonstrates that molecules used for treatment in other diseases may have better efficacy in sporotrichosis than traditional antifungals. Several molecules with anti-*Sporothrix* activity have been described. Recently, acylhydrazone molecules, which have low toxicity compared to current drugs, target vesicular transport, and cell cycle progression, have exhibited potent antifungal activity against *Sporothrix* spp. isolates.^29^ Molecules from Pathogen Box (Medicines for Malaria Venture, Switzerland) recently demonstrated that 80% of the studied isolates had growth inhibition by the compounds MMV102872 and iodoquinol,^11^ which initially had action against *Fonsecaea* spp. isolates.^10^ Sertraline, an antidepressant with *in vitro* activity against *Cryptococcus* spp., *Coccidioides* spp., and *Trichosporon* spp., has also recently been described as a fungicide for *S. schenckii.* ^30^

The MIC of *S. terebinthifolius* extracts for *S. brasiliensis*, and for the reference strains of *S. schenckii* and *S. globosa* were similar to those of many antifungals, such as azoles.^31^ *S. brasiliensis* is the main causative agent of zoonotic transmission in Brazil; therefore, we focused on the analysis of isolates of this species, obtaining inhibition not only for the reference strain, but also for all tested isolates.

Studies have shown that, in addition to *Candida tropicalis*,^32^ *S. terebinthifolius* extracts also have activity against *Candida albicans*,^33^ *Escherichia coli*, *Klebsiella pneumoniae*, *Proteus mirabilis*, *P. aeruginosa*, *Salmonella enteritidis*, *Staphylococcus aureus* ^34^ and *Leishmania amazonensis*.^18^ In *C. albicans*, the activity of *S. terebinthifolius* extracts may be related to cell wall formation.^32^ In *S. brasiliensis* and *S. schenckii*, cell wall composition is one of the factors influencing the susceptibility profile to traditional antifungal agents.^11^ ^,^ ^35^ Differences in this composition among *Sporothrix* species may help to explain the possible targets for *S. terebinthifolius* extracts, similar to the mechanism described for species of the genus *Candida*.

*In conclusion* - The results of this study revealed that the leaves of *S. terebinthifolius* have antifungal activity, probably due to chemical compounds such as flavonoids and terpenoids.

Literature has witnessed the great biological potential of *S. terebinthifolius*, in addition to the safety of its pharmacological use. Our results showed that the extract and fractions were active *in vitro* against *S. brasiliensis*. A larger sample of isolates from this and other species might reveal whether the effect of these extract and fractions is strain- or species-dependent. Therefore, the prospect is to continue with further investigation using *in vitro* and *in vivo* models, so that *S. terebinthifolius* can be used for the therapeutics of sporotrichosis in the future. The route of administration (oral or local), the appropriate formulation (cream, ointment, or capsule), whether alone or as adjuvant therapy, and whether it can be used in humans, cats, and dogs are questions to be answered in future investigations.
