# Supplementary material for: In vitro activity of Schinus terebinthifolius extract and fractions against Sporothrix brasiliensis
Source: Mem Inst Oswaldo Cruz. 2022 Sep 30;117:e220063. doi: 10.1590/0074-02760220063 (PMC9524759; doi:10.1590/0074-02760220063)
Supplement: Supplementary file 1 [file 1678-8060-mioc-117-e220063-s.pdf]

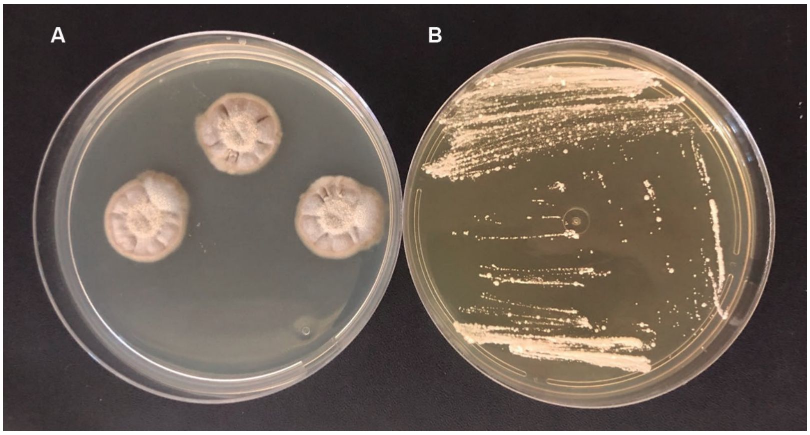

Fig. 1: representative macroscopy of *Sporothrix brasiliensis*. (A) Filamentous phase, cultured on Potato Dextrose Agar, at 25-30ºC, and (B) yeast phase, cultured on Brain Heart Infusion Agar, at 35-37ºC.

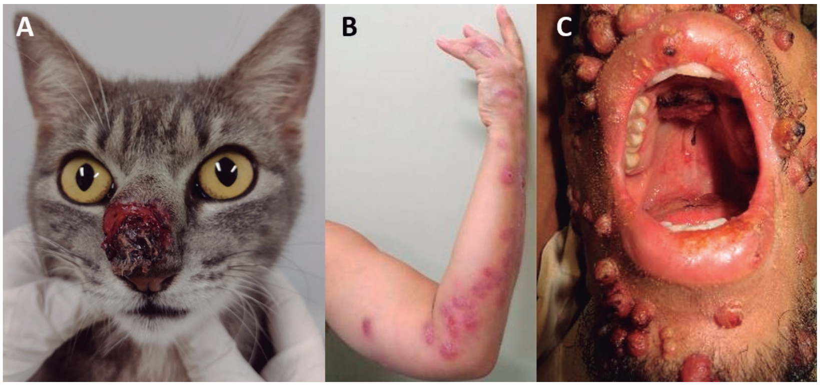

Fig. 2: clinical presentations of sporotrichosis. (A) Cat with a nodular-ulcerative lesion on the nose. (B) The classic lymphocutaneous form on the superior limb of a woman. (C) Disseminated papular and nodular-ulcerative lesions on the face and oral mucosa of a man with acquired immunodeficiency syndrome (AIDS).
Sources: (A) Laboratory of Clinical Research on Dermatozoonoses in Domestic Animals; (B-C) Laboratory of Clinical Research on Infectious Dermatology, Evandro Chagas National Institute of Infectious Diseases, Oswaldo Cruz Foundation.

CC BY
